# Us, them, and the others: Testing for discrimination amongst outgroups in a single‐piece nesting termite, *Zootermopsis angusticollis*

**DOI:** 10.1002/ece3.9901

**Published:** 2023-03-21

**Authors:** Rebecca F. B. Padget, Michael A. Cant, Faye J. Thompson

**Affiliations:** ^1^ Centre for Ecology and Conservation, College of Life and Environmental Sciences University of Exeter Cornwall UK; ^2^ Centre for Research in Animal Behaviour, College of Life and Environmental Sciences University of Exeter Exeter UK; ^3^ German Primate Center University of Goettingen Goettingen Germany

## Abstract

Recognition of group members is an important adaptation in social organisms because it allows help to be directed toward kin or individuals that are likely to reciprocate, and harm to be directed toward members of competing groups. Evidence in a wide range of animals shows that responses to outgroups vary with context, suggesting that cues to group membership also depend on the social or environmental context. In termites, intergroup encounters are frequent and their outcomes highly variable, ranging from destruction of a colony to colony fusion. As well as genetic factors, nestmate recognition in social insects commonly relies on cues that are mediated by environmental factors such as food source. However, single‐piece nesting termite colonies share nesting material and food source with rival colonies (their wood substrate serves as both). In principle, the shared environment of single‐piece nesting termite colonies could constrain their ability to identify non‐nestmates, contributing to some of the variation seen in encounters, but this has not been investigated. In this study, we raised incipient colonies of a single‐piece nesting termite, *Zootermopsis angusticollis*, on two different wood types and conducted behavioral assays to test whether nestmate discrimination can be constrained by common environmental conditions. We found that non‐nestmates elicited higher rates of identity checking and defense behavior compared to nestmates, but there was no effect of wood type on the strength of behavioral responses to non‐nestmates. We also found that one key cooperative behavior (allogrooming) was performed equally toward both nestmates and non‐nestmates. These findings offer no support for the hypothesis that common wood type constrains the nestmate recognition system of single piece nesting termites. We suggest that where groups encounter each other frequently in a common environment, selection will favor discrimination based on genetic and/or higher resolution environmentally mediated cues.

## Graphical abstract

Group member recognition is crucial in social evolution and cues to group identity are often derived from the environment. In single‐piece nesting termite species competing colonies live in and feed on the same log, which could potentially constrain nestmate recognition. We tested this by experimentally introducing non‐nestmate intruders raised on either the same or a different wood substrate and found non‐nestmates were not treated differently based on wood type. Nestmate discrimination mechanisms can operate even in a common environment and appear linked to environmental cues on a much finer scale than previously recognized.

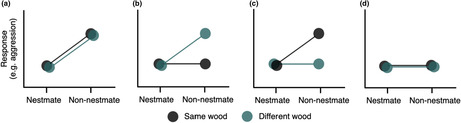

## 1 INTRODUCTION

The ability to target helping behaviors toward certain individuals and not others is thought to be central to the evolution of cooperation and altruism (Gadagkar, 1985; Hamilton, 1964). Recognition of group members allows individuals in cooperative social groups to direct their helping behavior toward individuals that are likely to be kin and/or to reciprocate, and to direct harming behavior toward individuals that are nonkin or direct competitors (West & Gardner, 2010). Outgroup members elicit predominantly agonistic responses, but the strength of response can vary with rival group identity and familiarity (Christensen & Radford, 2018; Temeles, 1994), and with context‐dependent factors such as population density (Thompson et al., 2017), location (Uematsu et al., 2019), and reproductive status (Golabek et al., 2012). Variation in ingroup responses to outgroups suggests that not all outgroups are treated as equal and that cues to group membership are, therefore, also likely to be modulated and dynamic.

In social insects, nestmates are characterized by a colony‐specific chemical signature determined by a combination of genetic and environmental cues (Adams, 1991; Vander Meer et al., 2019; van Zweden & d'Ettorre, 2010). In termites, cuticular compounds and the gut microbiome are potential sources of odor cues that individuals use to identify nestmates (Matsuura, 2001; Minkley et al., 2006; van Zweden & d'Ettorre, 2010). Cross‐fostering experiments in termites have shown that manipulation of cuticular compounds and the gut microbiome can alter responses to nestmates and non‐nestmates (Aguilera‐Olivares et al., 2016; Kirchner & Minkley, 2003; Matsuura & Nishida, 2001). As well as being influenced by genetic factors, both cuticular compounds and the gut microbiome can be influenced by environmental factors, such as diet (Chandler et al., 2011; Liang & Silverman, 2000). In single‐piece nesting termites, colonies are confined to live and feed within a single piece of wood in which the colony was founded and, as such, diet and local environment are shared amongst interacting colonies. These species therefore present a particularly interesting test case for environmental mediation of nestmate recognition. Confinement of the colony to a single nesting material and food source might limit the extent to which environmental factors cause nestmate recognition cues to diverge amongst interacting colonies, potentially leading to limitations on how precise nestmate recognition can be. These limitations could result in nestmate recognition errors, allowing non‐nestmates to join other colonies.

Colonies of termites are founded by a monogamous king and queen who produce a colony of diploid helpers. While some species are central‐place foragers, maintaining a nest and foraging distant resources, single‐piece nesting termites complete the entire life cycle of the colony within a single piece of dead wood. Thought to be the ancestral condition, around 19% of species are single‐piece nesting (Inward et al., 2007; Mizumoto et al., 2022). In these species, a high level of competition over the same nesting space and food resource results in frequent interactions between colonies that live at high densities (Thorne et al., 2003). During colony‐colony encounters, behavior toward non‐nestmates is often agonistic and can lead to the complete destruction of one of the colonies (Thorne et al., 2002). However, in some species (including single‐piece nesting species), interactions between colonies can be affiliative or cooperative (Cooney et al., 2016) and can result in the two colonies fusing and seemingly acting as one new colony (Howard et al., 2013; Johns et al., 2009; Thorne et al., 2003). While many termites have fully nonreproductive workers, when colonies of single‐piece nesting termites fuse, kings and queens from one colony are often killed and helpers (so called ‘pseudergates’) can begin to reproduce, conferring a direct fitness benefit on these new reproductives (Johns et al., 2009; Thorne et al., 2002). We do not yet understand the mechanisms that drive different outcomes of colony–colony encounters but developing our understanding of nestmate recognition—in particular, which features are conserved or divergent across the phylogeny—could help explain the variation in behavioral responses of termites to non‐nestmates both within and amongst taxa. Single‐piece nesting termites make a particularly interesting test case for this because interacting colonies share the same nesting environment, meaning that environmentally mediated recognition cues are likely to be more similar in the interacting colonies than they are, for example, when central place foraging colonies meet.

If environmentally mediated cues were a strong component of nestmate recognition in single‐piece nesting termites, we might expect the strength of response to a non‐nestmate to be proportional to the difference in the type of environment in which the two interacting colonies were found. Over a small spatial scale (that of the log resource), environmental differences might stem from, for example: variation in the decomposition of the wood, exposure to sunlight or shade, or whether parts of the log are in contact with, partially buried in, or raised off the ground. Different species of wood are also likely to differ in these factors, containing different microbial communities, decomposing at different rates, and responding differently to environmental factors. Although two colonies of single‐piece nesting termites found in different species of wood would not meet each other and interact in nature, if termites do acquire recognition cues from their environment, these are likely to be most divergent amongst colonies on different species of wood. The limited environment that these termites experience (despite their initial capacity to nest in different wood types) provides an experimental opportunity to test the effect of environmentally mediated cues on nestmate recognition where these cues are likely to differ to the greatest extent.

We tested whether single‐piece nesting termites responded differently to non‐nestmates who were raised on the same wood type as them, compared to those raised on a different wood type. This would allow us to determine whether nestmate recognition was constrained for individuals reared on the same type of wood during encounters with individuals from different colonies. We manipulated the environment of incipient colonies of the single‐piece nesting termite, *Zootermopsis angusticollis* (Hagen), rearing colonies on two different types of wood substrate. We then conducted assays of the behavioral responses of pseudergates to an introduced non‐nestmate (or nestmate control).

**FIGURE 1:**
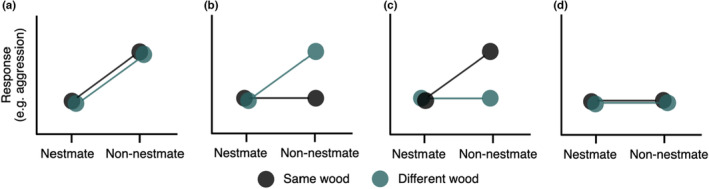
Schematic of a priori predictions of potential outcomes considered before the experiment. Each figure illustrates a potential outcome of newcomer introductions based on four related hypotheses: (a) termites discriminate non‐nestmates, but not between different types of non‐nestmate (nest environment plays no role); (b) termites discriminate non‐nestmates but are less able to discriminate those raised on the same wood type as them (similar nest environment constrains discrimination); (c) termites discriminate non‐nestmates but are less able to discriminate those raised on a different wood type to them (similar nest environment facilitates discrimination); and (d) termites do not discriminate non‐nestmates (relative to the nestmate control). Figures represent our predictions and are not based on observed data.

We made predictions based on four related hypotheses: (a) termites discriminate non‐nestmates from nestmates, but not between non‐nestmates reared on different wood types because the environment does not play a primary role in nestmate recognition; (b) termites discriminate non‐nestmates but are less able to discriminate those raised on the same wood type as them because they use environmentally mediated cues and discrimination ability is (monotonically) related to cue difference; (c) termites discriminate non‐nestmates but are less able to discriminate those raised on a different wood type to them because although they might use environmentally mediated cues, there has been no selection on interactions involving termites with extreme cue divergence because termites on different wood types would not meet in nature; and (d) termites do not discriminate non‐nestmates from nestmates (Figure 1). *Zootermopsis* termites show a more aggressive response to heterospecifics than to non‐nestmate conspecifics (Haverty & Thorne, 1989). We, therefore, assume that this will also be the case in encounters with different types of conspecifics, and that level of aggression is reflective of termites' ability to classify individuals as non‐nestmates, and predict that the response to non‐nestmates will be aggressive.

## 2 METHODS

### 2.1 Termite collection and maintenance

Stock colonies used to find all incipient colonies used in the experiment were established by collecting whole natural *Z. angusticollis* colonies from Redwood Regional Park (37°48′49″ N, 122°09′57″ W). Whole, natural colonies were collected under permit from East Bay Regional Parks and the California Department of Fish and Wildlife, and imported under license from the UK Animal and Plant Health Agency to the Centre for Ecology and Conservation, University of Exeter, UK.

Each stock colony was housed in a 35‐L plastic box containing a mixture of their native wood and birch (*Betula*; Cole et al., 2018; Cole & Rosengaus, 2019). We used these stock colonies to establish the 39 incipient colonies used in this experiment. Eighteen known stock colonies were used to establish 31 of the incipient colonies used. For eight incipient colonies, the stock colony of one or both of the alates was unknown. Thirty‐four of the 39 incipient colonies were founded between 2018 and 2019 (supplied by R. Rosengaus' laboratory at Northeastern University, USA). The other five incipient colonies used were established in August 2019 at the Centre for Ecology and Conservation, University of Exeter, UK.

Incipient colonies were founded by pairing de‐winged, unmated alates collected during dispersal events from laboratory stock colonies. Termites paired to find these incipient colonies were collected from laboratory stock colonies while they still had wings, and were de‐winged by experimenters, so it is assumed that they were unmated prior to collection. These pairs were then allowed to raise an incipient colony in a 50 mm by 15 mm round Petri dish lined with moistened cellulose filter paper and some small pieces of European silver birch wood (*Betula pendula*; as described in Cole et al., 2018 and Cole & Rosengaus, 2019). When incipient colonies had developed ~5 larvae and a soldier, they were transferred, along with their wood, to a larger (100 mm), square Petri dish. Of the 39 incipient colonies used in the trials, 24 were founded by inbreeding alates from the same stock colony, eight by outbreeding alates from different stock colonies and seven were founded by reproductives of unknown origin. Incipient colonies were between 7 and 19 months old and contained at least seven individuals, including at least one soldier and two reproductives; maximum colony size was ~50 individuals. None of the incipient colonies contained secondary reproductives or reproductive soldiers.

After they were established, we continued to provide silver birch ad libitum throughout development to 25 of the incipient colonies; 14 incipient colonies were transferred onto sycamore (*Acer pseudoplatanus*) at least 1 month prior to trials, typically during transfer to the larger Petri dish, and supplied with sycamore ad libitum thereafter. Neither birch nor sycamore are natural nesting woods for *Z. angusticollis*, but both are hardwoods, on which termites can raise a colony (established by prior trials of both woods as a food source at the Centre for Ecology and Conservation). The birch wood used was collected from the local area in Penryn and all came from a single tree; the sycamore also came from a single sourcing of wood chips, but it cannot be guaranteed that all sycamore came from the same tree. Using single wood sources controlled for additional differences in the wood, for example, different growth rates in different regions.

All colonies were kept in the dark in a controlled environment at 22°C and 85% humidity. Moisture was introduced by initially soaking the wood substrate and a small amount of cellulose filter paper (~2 × 3 cm) in distilled water, and was maintained by spraying colonies with distilled water approximately twice weekly.

### 2.2 Behavioral assays

**FIGURE 2:**
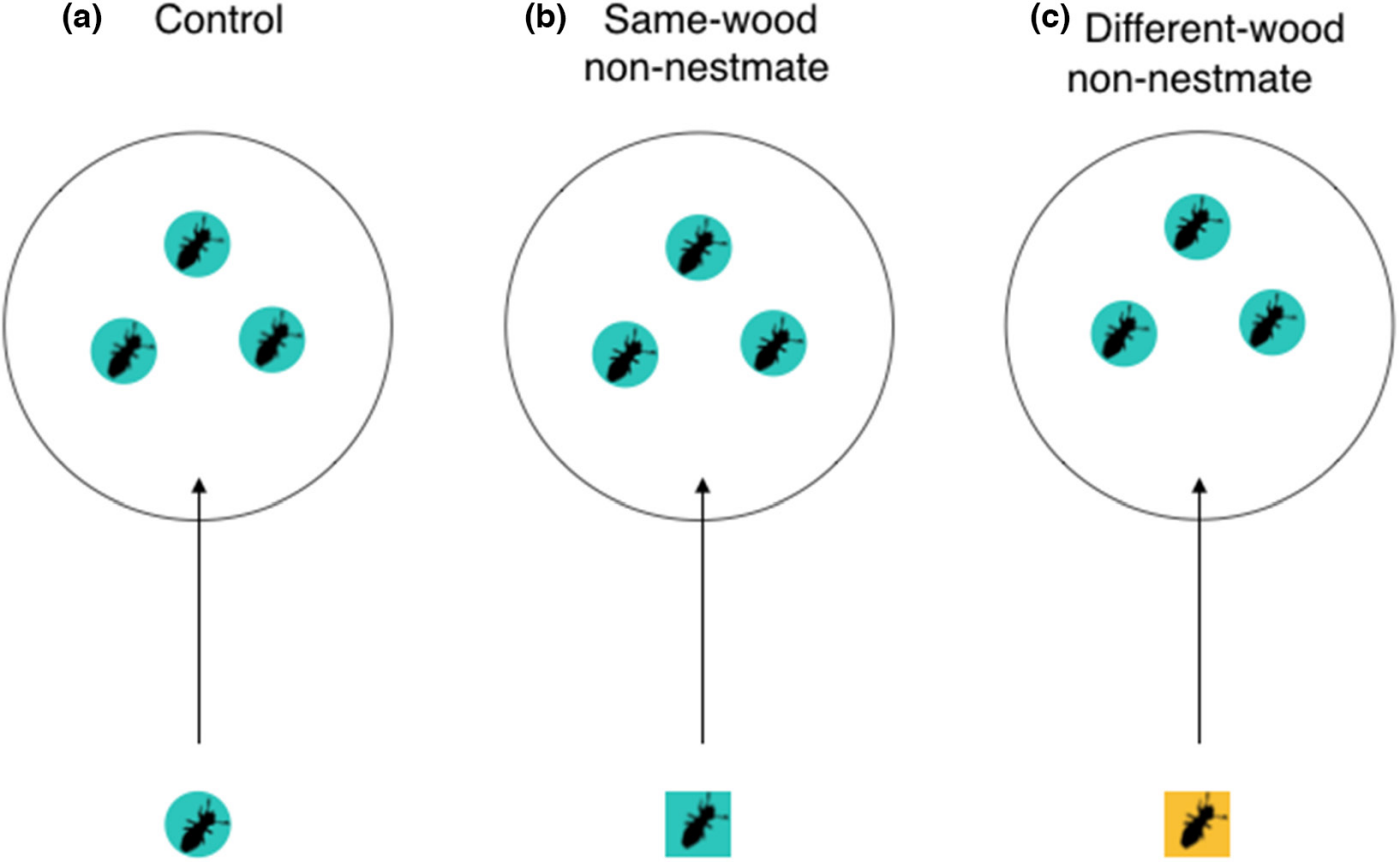
Schematic of an example trial set up. Shapes represent colony identity; colors represent wood type. An individual was introduced to a group of three workers from either its own (a) or a different (b, c) colony. If it was introduced to individuals from a different colony, they could be raised on either the same (b) or a different (c) wood type.

Experiments were carried out over two trial days in February and March 2020 at the Centre for Ecology and Conservation, University of Exeter, UK. Trials were conducted in batches of three replicates at a time. For each replicate, three termite pseudergates from an incipient colony were placed into an uncovered 50‐mm round Petri dish containing a cut‐to‐size circle of dry filter paper. Groups were left to settle for 2 min under red light (provided by two bulbs positioned around 1.5 m above the Petri dishes) in an otherwise dark room. A fourth pseudergate, a newcomer, was then introduced and allowed to interact for a 3‐min ‘trial period’. While 3 min is a short period of time, it was sufficient to allow the four individuals opportunity to interact and observe the immediate response to the newcomer. The newcomer was one of: a non‐nestmate raised on the same type of wood (birch newcomer into birch group = 10; sycamore newcomer into sycamore group = 10; total = 20); a non‐nestmate raised on a different type of wood (sycamore newcomer into birch group = 9; birch newcomer into sycamore group = 8; total = 17); or a nestmate control (birch = 14; sycamore = 3; total = 17; Figure 2). Individuals were not marked since the observer could visually keep track of the introduced termite without the need for marking. We did not note the sex of the termites because male and female pseudergates are similar in both morphology and behavior. Videos were recorded for the whole 5‐min settling and trial time using a Sony HDR‐CX240E or a Sony HDR‐PJ330 video camera mounted on a tripod roughly 30 cm above each Petri dish; each camera videoed a single Petri dish. Of the 39 unique colonies that were used in the trials, 16 were used on both trial days, which were roughly 2 weeks apart. On each occasion workers were collected at random meaning that, since workers were returned to their natal colony at the end of each trial, some workers might have been used in two trials.

Termites were not size‐matched, so the size ratio of the newcomer was included in analyses to account for effects that a larger or smaller newcomer might have on termite responses. To calculate the size ratio, we measured the body length of each termite (in millimeters) post hoc from the videos and divided the newcomer's length by the mean length of individuals in the interacting group (excluding the newcomer). Thus, a size ratio >1 means the newcomer was larger than the mean of the group, and vice versa if the size ration was <1.

The relatedness of the newcomer to the group of termites was unknown because we did not know the relatedness of the stock colonies to each other, and we did not know which stock colonies all incipient colonies were established from. Of the 28 non‐nestmate pairings for which we knew the parental (alate) stock colonies, four shared a parental stock colony and 24 did not; for nine pairings, we did not know the parental stock colonies; and 17 pairings were controls. There were therefore insufficient data to establish whether genetic relatedness had an effect on behavioral response to newcomers in this experiment.

Videos were coded using Behavioral Observation Research Interactive Software (BORIS; Friard & Gamba, 2016). Newcomer identity (nestmate or non‐nestmate) was not known to the observer during data collection. To account for small discrepancies in introduction and filming times, data were collected from the last 2 min 30 s of each trial period. We recorded either the proportion of the observation time or the number of times that individuals directed behaviors associated with affiliation (allogrooming, antennation, trophallaxis), aggression (biting, butting), and defense (recoiling) to the newcomer (Table 1). For affiliative behaviors, we recorded the proportion of time that individuals directed these behaviors toward the newcomer termite. For aggressive and defensive behaviors, which were shorter in duration than affiliative behaviors and occurred in discrete events, we recorded the number of times that individuals directed these behaviors toward the newcomer.

**TABLE 1:** Ethogram of behaviors observed during video coding.

Behavior	Description	Data type	Reference
**Allogrooming**	**Affiliative. Uses mandibles on recipient's body or head such as not to cause injury or fleeing**	**Proportion time newcomer was allogroomed**	Korb (2008), Korb et al. (2012) and Zhukovskaya et al. (2013)
**Antennation**	**Affiliative. Moves antennae over recipient's head, body or antennae**	**Proportion time newcomer was antennated**	Korb et al. (2012)
**Recoiling**	**Defensive. Sudden jump back from another termite, often following short antennation period**	**Number of times residents performed behavior toward newcomer**	Šobotník et al. (2008)
Biting	Aggressive. Uses mandibles on another termite's head or body and causes injury or fleeing response	Number of times newcomer was bitten	Ishikawa and Miura (2012)
Butting	Aggressive. A dominance behavior involving quick vibrations of the whole body	Number of times resident individuals performed butting	Korb et al. (2012)
Trophallaxis	Affiliative. Mouth to mouth or mouth to anus contact with another termite	Proportion time residents engaged in trophallaxis (gave or received) with newcomer	Crosland et al. (1997), but see Korb and Schmidinger (2004)

*Note*: A description of the behavior and how it was measured are provided. Behaviors in bold are those that we observed sufficiently to carry out statistical analyses.

### 2.3 Statistical analysis

Of the behaviors measured, only three (allogrooming, antennation, and recoiling) occurred sufficiently for statistical analysis. Allogrooming and recoiling were observed in 23, and antennation was observed in 53 of the 54 replicates. Trophallaxis involving the newcomer occurred in four replicates (three mouth‐to‐mouth, one mouth‐to‐anus where the newcomer was the recipient); biting of the introduced individual was recorded in one replicate (biting by the introduced individual was recorded in one other replicate); butting was never recorded. Trophallaxis, biting, and butting were, therefore, excluded from statistical analyses.

Data for all analyzed behaviors contained a high proportion of zeroes, and assumptions of normality and homoscedasticity of residuals for standard linear models were not met, even after transformation of the data. Therefore, we used Markov chain Monte Carlo (MCMC) to fit generalized linear mixed effects models (GLMMs) to (untransformed) data under a Bayesian framework, using scale‐adjusted, weakly informative priors ($\beta_{i}∼N\left(0,2.5\right)$). We fitted separate models for each behavior. We assumed a zero‐inflated beta distribution for the data in the allogrooming and antennation models, following guidelines in Douma and Weedon (2019) for analysis of continuous proportions, and a negative binomial distribution for the recoiling model to account for overdispersion caused by zero inflation. We included newcomer identity (nestmate or non‐nestmate) and the interaction between newcomer identity and wood type (same wood or different wood) as fixed effects in each model. Including a main effect of wood type would not be meaningful because all nestmate controls were necessarily raised on the same wood type as the newcomer, so we included wood type only in the interaction term with newcomer identity. We included size ratio as an additional fixed effect. Resident colony ID (a factor with 39 levels) was included as a random effect to account for the colonies that were used twice. Filming batch (1–12) and trial day (a factor with two levels) were also included as random effects to account for any temporal variation in environmental factors that might have altered behavior within or between trial days, respectively.

To investigate the effect of each fixed effect on the response, we calculated inclusion Bayes factors for each fixed effect. The inclusion Bayes factor is the average ratio of posterior probabilities of all the models including the fixed effect of interest over all those that do not. Models compared were those produced by dropping each fixed effect in turn. Models that contained the interaction term (between newcomer identity and wood type) also always contained the main effect of wood type because all controls (nestmates) were necessarily reared on the same wood type. We interpret inclusion Bayes factors as evidence for an effect of each fixed effect in line with convention set out in Jeffreys (1961) such that: a Bayes factor between 1 and 3 is considered ‘weak’ evidence in favor of the alternative; between 3 and 10 is considered ‘moderate’ evidence; and between 10 and 30 is interpreted as ‘strong’ evidence (Lee & Wagenmakers, 2014). A Bayes factor <1 is considered evidence in favor of the null: between 1 and 1/3 is weak evidence in favor of the null; between 1/3 and 1/10 moderate; and between 1/10 and 1/30 is considered strong evidence in favor of the null. We report the coefficient estimate as the median of the posterior distribution, and we report the 89% highest density interval (HDI) of the posterior coefficient distributions from the full model to describe the uncertainty around the coefficient estimate.

Since a large proportion of our incipient colonies were established from inbred pairs of alates from the same stock colony, we conducted an additional post hoc analysis to identify whether a group's inbreeding status played a role in their behavioral response. For the three behaviors analyzed (allogrooming, antennation, and recoiling), we added an additional fixed term to each model: inbreeding status, a two‐level factor describing whether the incipient colony was established using (inbred) alates from the same stock colony or (outbred) alates from different stock colonies. There were seven incipient colonies (involved in 11 trials) for which the origin of one or both of the alates was unknown; trials involving these colonies were therefore excluded from this additional analysis (sample size for this additional analysis was therefore *N* = 43 trials). To determine whether inbreeding status helped to explain our observed data, we calculated the Bayes factor in favor of the model including inbreeding status over the model without including inbreeding status (re‐run on the same data as the inbreeding model to meet the assumptions for calculating Bayes factors). We found weak evidence of an effect of inbreeding status on allogrooming, but we found no evidence that inbreeding status had an effect on antennation or recoiling. Because this analysis required removing 11 replicates, and because there was no evidence of an effect of inbreeding, we report models that do not include inbreeding status in the main text, and report our inbreeding analysis here in Appendix S1. This allows us to not only maintain statistical power from the full sample size but also take into account the potential effect of inbreeding on allogrooming in our conclusion.

Analyses were carried out in R3.6.1 (R Core Team, 2022) using the ‘brms’ library (Bürkner, 2018) for the zero‐inflated beta models, and the ‘rstanarm’ library (Brilleman et al., 2019) for the negative binomial models. We used bayestestR to calculate Bayes factors (Makowski et al., 2019), and plots were generated using bayesplot (Gabry et al., 2019) and ggplot2 (Wickham, 2016). Code for all analysis is available at: https://github.com/beckypadget/nestmate_recognition.

## 3 RESULTS

**FIGURE 3:**
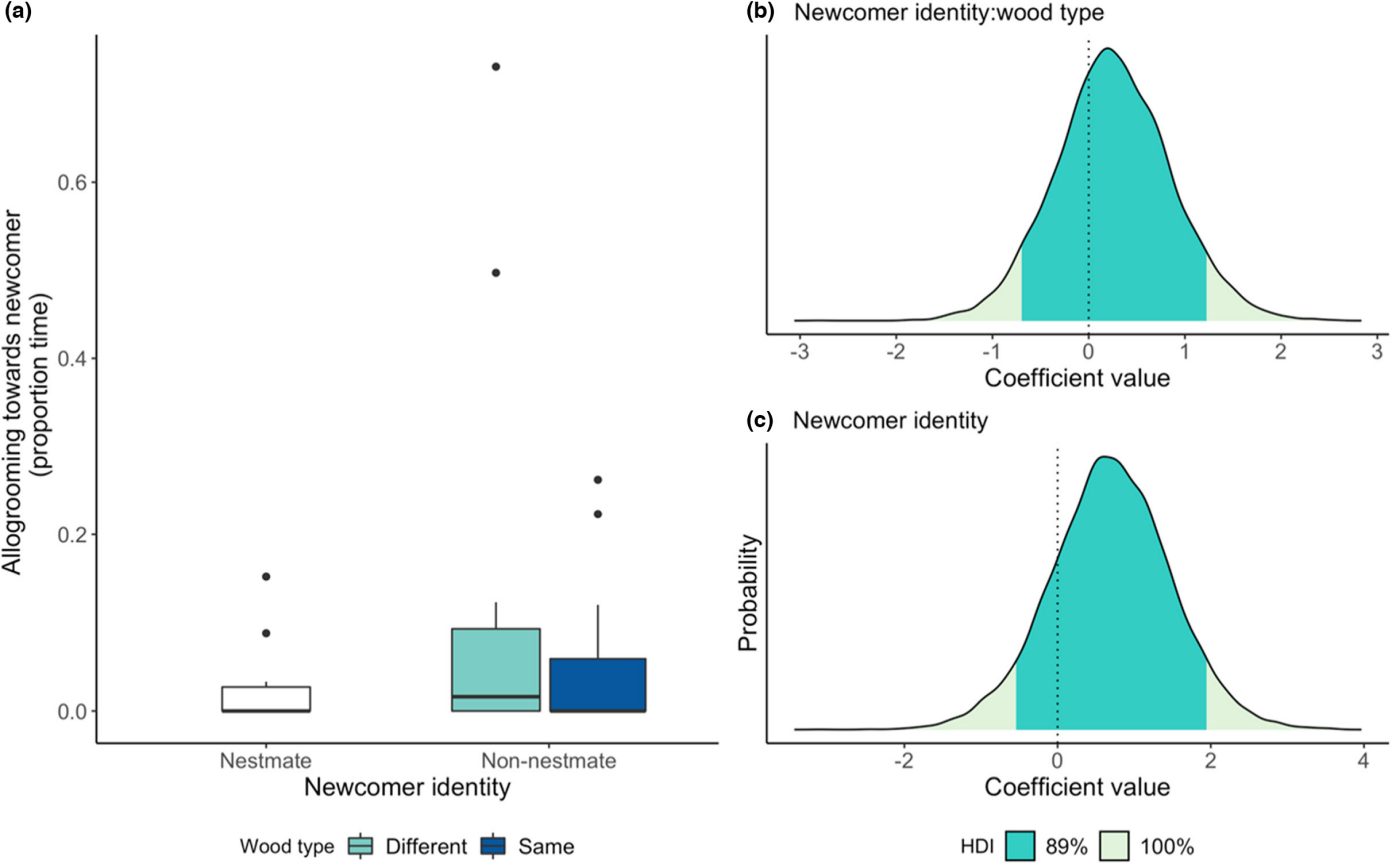
The effects of newcomer identity and wood type on allogrooming. Raw data of the effect of newcomer identity and wood type on allogrooming (a) and the posterior coefficient distribution for the interaction between newcomer identity and wood type interaction term (b), and newcomer identity term (c) from the full model. Dark green shaded areas on coefficient distribution plots (b, c) show the 89% highest density interval (HDI); dotted lines at zero show where zero lies within the posterior coefficient distribution. For both the interaction term and the main effect of newcomer identity, the zero coefficient line falls well within the 89% HDI region, indicating that there is no effect of either newcomer identity or wood type on allogrooming behavior.

**FIGURE 4:**
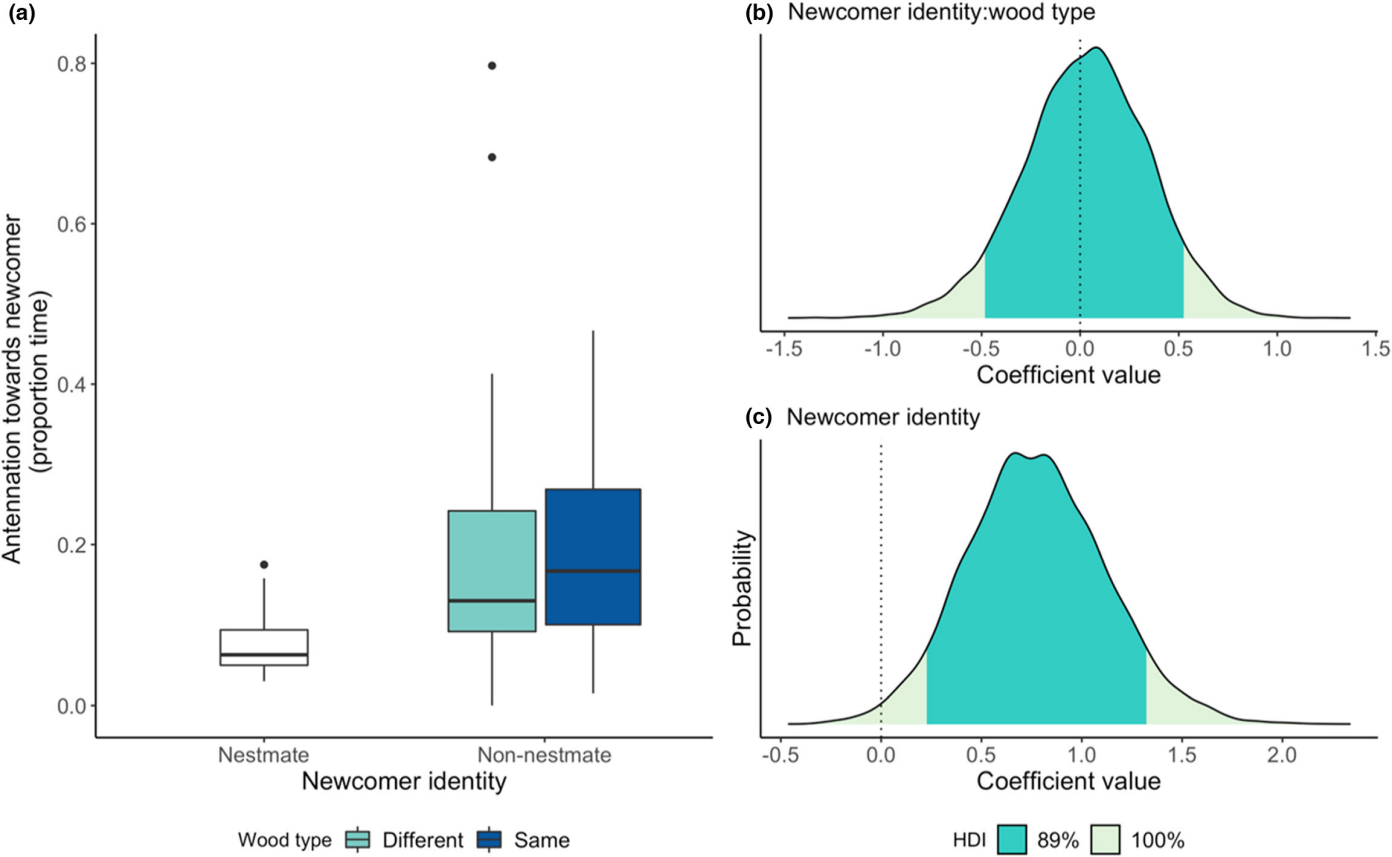
The effects of newcomer identity and wood type on antennation. Raw data of the effect of newcomer identity and wood type on antennation (a) and the posterior coefficient distribution for the interaction between newcomer identity and wood type interaction term (b), and newcomer identity term (c) from the full model. Dark green shaded areas on coefficient distribution plots (b, c) show the 89% highest density interval (HDI); dotted lines at zero show where zero lies within the posterior coefficient distribution. The zero coefficient line falls outside of the 89% HDI region, indicating that there is an effect of newcomer identity on antennation behavior.

**FIGURE 5:**
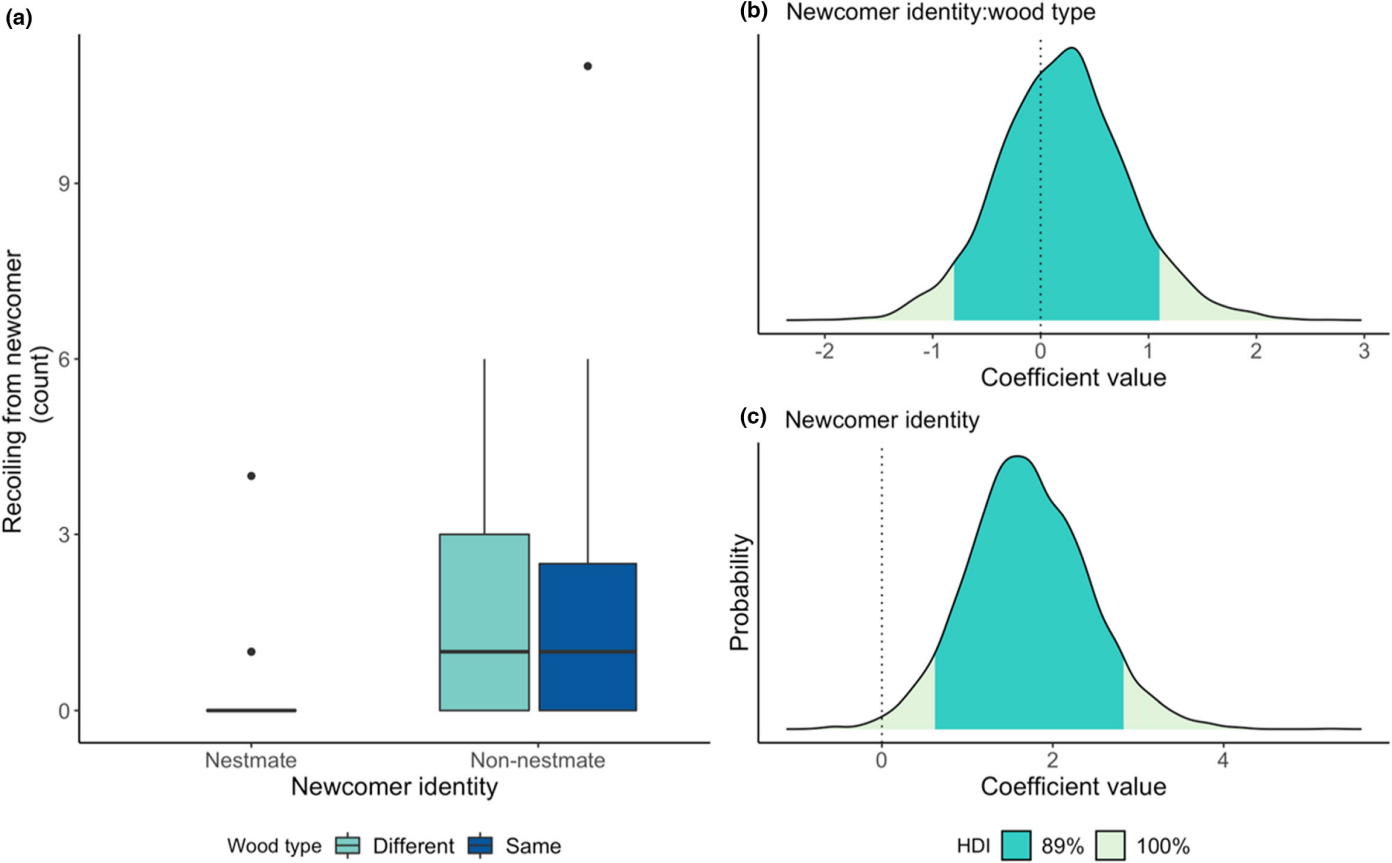
The effects of newcomer identity and wood type on recoiling. Raw data of the effect of newcomer identity and wood type on recoiling (a) and the posterior coefficient distribution for the interaction between newcomer identity and wood type interaction term (b), and newcomer identity term (c) from the full model. Dark green shaded areas on coefficient distribution plots (b, c) show the 89% highest density interval (HDI); dotted lines at zero show where zero lies within the posterior coefficient distribution. The zero coefficient line falls outside of the 89% HDI region, indicating that there is an effect of newcomer identity on recoiling behavior.

Termites did not respond differently to non‐nestmates reared on a different wood type (newcomer identity x wood interaction, allogrooming: BF_inclusion_ = 0.26, estimate = 0.25, 89% HDI = −0.70, 1.22; antennation: BF_inclusion_ = 0.21, estimate = 0.03, 89% HDI = −0.48, 0.52; recoiling: BF_inclusion_ = 0.44, estimate = 0.2, 89% HDI = −0.81, 1.10; Figures 3, 4, 5). Irrespective of wood type, termites allogroomed nestmates and non‐nestmates equally (newcomer identity, allogrooming: BF_inclusion_ = 0.36, estimate = 0.69, 89% HDI = −0.54, 1.95; Figure 3a,c). However, non‐nestmates received, on average, 18 seconds (12% of the 150‐second observation time) more antennation than nestmates (newcomer identity, antennation: BF_inclusion_ = 3.5, estimate = 0.77, 89% HDI = 0.23, 1.32; Figure 4a,c). Similarly, non‐nestmates elicited higher levels of recoiling than nestmates did, around 1.8 more recoiling events (newcomer identity, recoiling: BF_inclusion_ = 11.8, estimate = 1.7, 89% HDI = 0.62, 2.82; Figure 5a,c).

**FIGURE 6:**
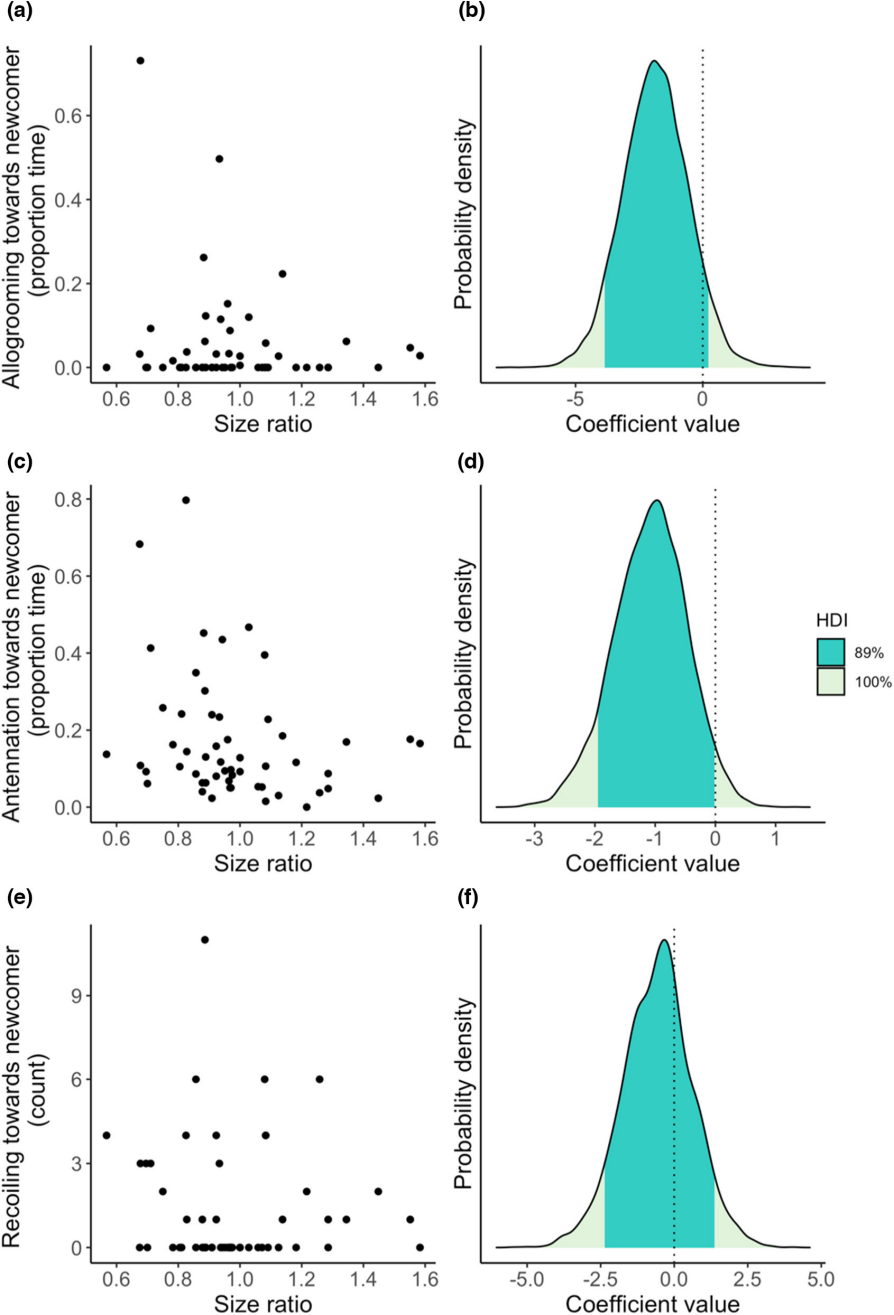
The effects of relative newcomer size on allogrooming, antennation, and recoiling. Raw data of the effect of the size ratio of newcomer, and the posterior coefficient distribution for allogrooming (a, b), antennation (c, d), and recoiling (e, f). Dark green shaded areas on coefficient distribution plots (b, d, f) show the 89% highest density interval (HDI); dotted lines at zero show where zero lies within the posterior coefficient distribution. For allogrooming and antennation, the zero coefficient line falls at the 255 boundary of the 89% HDI region indicating (when considered together with the Bayes factor) that there is a weak effect of relative size on allogrooming and antennation behavior. In contrast, there is no effect on recoiling behavior.

The inclusion Bayes factor indicated weak evidence that relative size had a negative effect on allogrooming: newcomers that were relatively larger than resident termites received less allogrooming than smaller newcomers (size, allogrooming: BF_inclusion_ = 3.1, estimate = −1.9, 89% HDI = −3.86, 0.22; Figure 6a,b). A 10% increase in relative size from the median size ratio resulted in a 0.75 s reduction in allogrooming. However, this effect was only apparent when inbreeding status was not in the model (Appendix S1), suggesting that this should be interpreted with caution. Relative size also had a weak effect on antennation, with a 10% increase from the median size ratio leading to a 1.1 second reduction in antennation (size, antennation: BF_inclusion_ = 1.2, estimate = −1.08, 89% HDI = −1.95, −0.02; Figure 6c,d). However, there was no effect of size on recoiling (size, recoiling: BF_inclusion_ = 0.12, estimate = −0.5, 89% HDI = −2.36, 1.37; Figures 6e,f).

## 4 DISCUSSION

If termites rely strongly on environmentally mediated nestmate recognition cues, non‐nestmates from a different environment should elicit a different response to those from the same environment. Contrary to this, we found that non‐nestmates elicited a similar response regardless of whether they were reared on the same or a different wood type (a proxy for environmental dissimilarity) as the group to which they were introduced. This result suggests that broad‐scale diet might not be a dominant driver of nestmate recognition in single‐piece nesting termites, a group in which colony–colony competition can occur frequently and between colonies reared in a common environment. Termites in our experiment did respond differently to non‐nestmates compared to nestmate controls—they were not more aggressive, but termites recoiled from and antennated toward non‐nestmates more than nestmates. Interestingly, we found evidence that nestmates and non‐nestmates received equal allogrooming—a behavior associated with cooperation. We suggest that frequent colony fusion in this group might select for recognition of out‐group members without the aggression typically associated with intergroup encounters.

The lack of discrimination between the two different out‐groups (same‐wood and different‐wood) in this experiment might be explained by mechanistic constraints on diet‐mediated cue variation in single‐piece nesting termites. While there is a direct link between diet and gut microbiome or diet and cuticular hydrocarbons in some insect taxa (Blomquist et al., 1987; Blomquist & Jackson, 1973), the mechanisms that facilitate diet‐based variation in these aspects of physiology are likely to be more limited in single‐piece nesting termites because they feed exclusively on damp wood. This diet requires a stable community of cellulose‐digesting gut microbiota, meaning that single‐piece nesting termites might be less tolerant of major fluctuations in gut microbiome. Similarly, species whose cuticular hydrocarbons are greatly affected by their diet are largely insectivorous, and their dietary hydrocarbons form direct precursors in endogenous hydrocarbon production (Liang & Silverman, 2000). Because they do not have a source of dietary hydrocarbons, this pathway is unlikely to be available to single‐piece nesting termites. Therefore, the extent to which diet could influence nestmate recognition cues might be limited to extreme or longer term differences in diet. Here, we found no evidence that extreme differences in diet (different wood type) affected nestmate recognition. However, the field would benefit from a more detailed mechanistic understanding of the development of nestmate recognition over time on both an individual and colony level.

**FIGURE 7:**
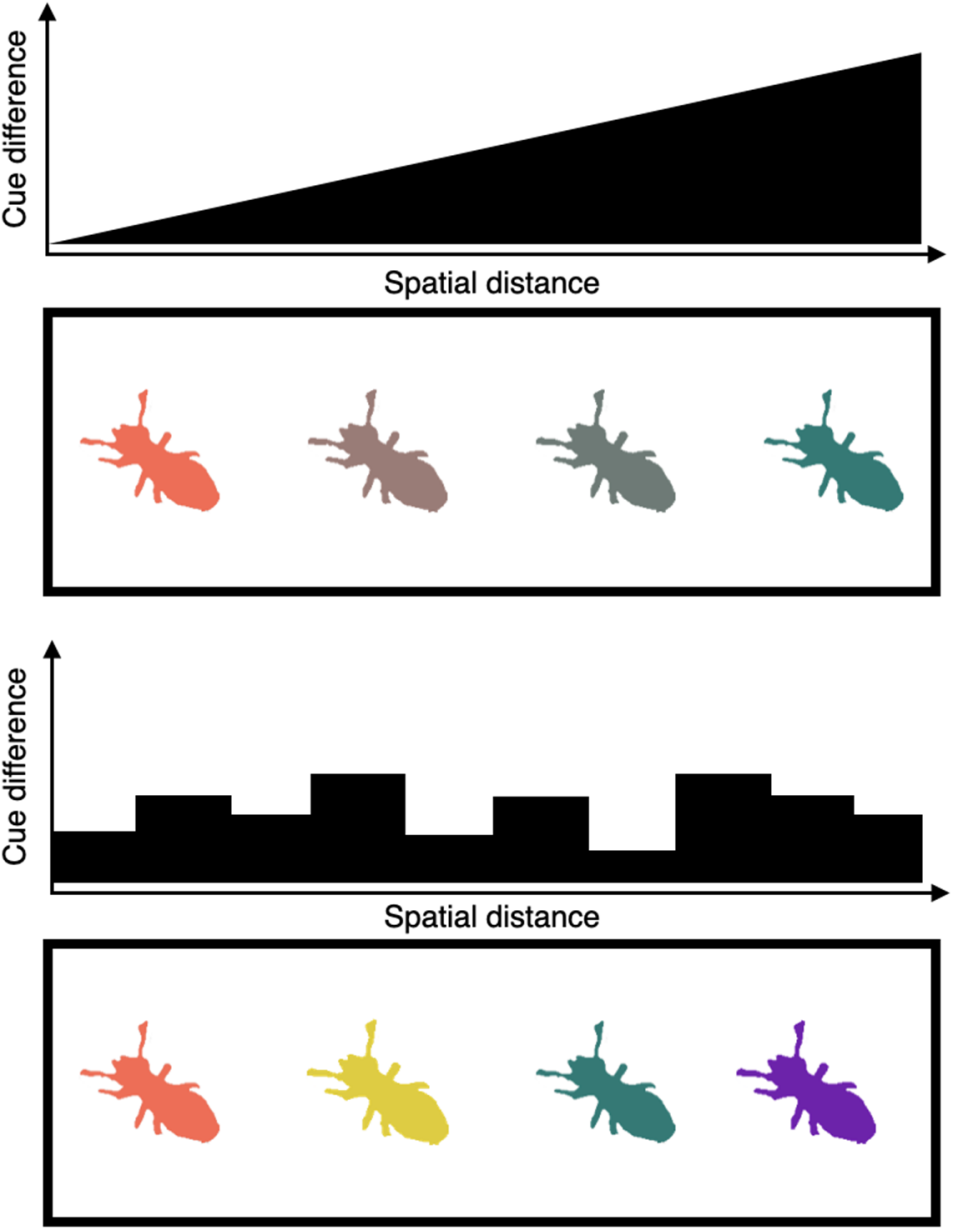
Schematic describing the difference between macro‐ (top) and microenvironmental (bottom) mediation of nestmate recognition cues, a potential explanation for our results. If nestmate recognition cues (represented by colors in this schematic) are mediated by broad‐scale environment, we might expect this to occur along a gradient such that colonies further apart have more diverging cues than those closer together (top diagram). However, microenvironmental features (bottom diagram), are unlikely to cause monotonic divergence with spatial distance in the same way, meaning that colonies next to each other differ just as much as colonies far away from each other, potentially resulting in outcomes similar to the ones we observed in our experiment.

Alternatively, nestmate recognition cues might depend on environmental mediation at a finer scale than that of wood type. Cues could vary with biotic and abiotic microenvironmental features, for example, fungal growth or humidity, that are heterogeneously distributed within a single piece of wood. If nestmate recognition cues vary with the microenvironment at this scale, then cue variation amongst individuals on the same piece of wood is likely to be higher than that between those on different wood types (Shellman‐Reeve, 1994; Figure 7). This could lead to the results that we observed in this experiment. Variation in recognition cues could also occur within colonies (Gordon et al., 2020), meaning that some level of tolerance or plasticity might be built in to nestmate discrimination to avoid inappropriate aggression toward nestmates, which could be costly (Faria & Gardner, 2020). How termites overcome this within‐colony variation in encounters where aggression is observed requires further study.

Previous research has found evidence that some higher termites are more aggressive to neighbor colonies than distant ‘stranger’ colonies (the nasty neighbor effect: Dunn & Messier, 1999; Kaib et al., 2002). Our finding that the response to non‐nestmates was unaffected by the newcomer colony's wood type suggests that colony substrate might not play a primary role, particularly in single‐piece nesting termites. However, colonies of single‐piece and foraging termites do appear to respond differently to non‐nestmates under different circumstances in both natural and experimental encounters. Colony–colony encounters can result in aggression, avoidance, or even peaceful colony fusion (Korb & Roux, 2012). Experimental work to investigate termite responses to other potential sources of cue variation amongst interacting non‐nestmates (e.g., kinship or other environmental factors) will be important for understanding the mechanistic processes underlying the variation in social outcomes that we observe in this group. Meanwhile, the application of theoretical models of intergroup conflict, particularly those that incorporate different types of outgroups (e.g., neighbors and strangers), are likely to provide insight into the adaptive value of plasticity in response to out‐group members.

Our observations of allogrooming (a behavior often interpreted as cooperative in some contexts) along with a lack of aggression toward non‐nestmates was unexpected but not unprecedented (Aguero et al., 2020; Cooney et al., 2016; Pan et al., 2006). High relatedness between colonies could result in non‐nestmates sharing similar heritable nestmate recognition cues, despite potential differences in environmentally mediated cues. These potentially conflicting cues could result in unfamiliar individuals eliciting cooperation as well as behaviors associated with identity checking and defense (antennation and recoiling, respectively; Šobotník et al., 2008; Thompson et al., 2020). All our stock colonies were collected from the same region. There is evidence of strong genetic differentiation in this population (Booth et al., 2012), but we do not know the genetic relatedness of the stock colonies from which our incipient colonies were founded. It is possible, therefore, that some inherited cues were shared amongst the colonies that we tested, which might have led to behavior that was uncharacteristically unaggressive. Alternatively, the cooperative behavior that we observed directed toward non‐nestmates could have been driven by fitness benefits that pseudergates—which are reproductively totipotent—might gain from colony fusion. After colony fusion, kings and queens are often killed and some pseudergates molt into secondary reproductives, thus gaining direct fitness benefits from the presence of non‐nestmate helpers within the natal colony (Howard et al., 2013; Johns et al., 2009; Korb & Roux, 2012). These benefits of cooperation with non‐nestmates could explain why termites allogroom non‐nestmates, despite apparently perceiving them as unfamiliar.

While relatedness between colonies might reduce aggression, an elevated level of relatedness within a colony, for example, due to inbreeding, might lead to increased aggression between colonies if nestmate recognition cues are genetically inherited and can become more strongly diverged between inbred groups. However, when we included inbreeding in our analysis, there was little evidence that it contributed to the behavior that we observed. Inbreeding is observed in natural termite colonies, both during colony founding by related alate kings and queens (Booth et al., 2012), and when secondary reproductives breed within a colony (Howard & Thorne, 2011). Further investigation into the effect of relatedness (both between colonies and within a colony) on nestmate recognition is required to better understand the impact of genetically mediated (dis)similarity in nestmate recognition cues in termites and its interaction with environmentally mediated cues.

We acknowledge that while we implemented measures to reduce disturbance (e.g., using red light, and allowing a settling period before observation), this experimental design likely caused some level of disruption to the termites, and this might have affected the range and frequency of behaviors that we observed. The impact of experimental design is evidenced in previous nestmate recognition experiments: trials in which termites are introduced in the novel environment of an open Petri dish (as in our experiment) tend to report lower rates of aggression than similar studies in which colonies are allowed to meet in more naturalistic enclosed settings (Cornelius & Osbrink, 2009; Messenger & Su, 2005, e.g. Cooney et al., 2016; Delphia et al., 2003; Haverty & Thorne, 1989; Howard et al., 2013; Johns et al., 2009; Thorne, 1982). The social context could also be important in determining behavior toward out‐group members—pseudergates appear to show greater levels of aggression in the presence of the reproductives than when reproductives are absent, as they were in our experiment (Ishikawa & Miura, 2012; Konishi & Matsuura, 2021). Similarly, the short period of time for which we observed the interactions in this experiment is unlikely to provide a complete picture of colony–colony interactions. Previous experiments have shown that while there can be little aggression over short periods, there can be high mortality when colonies are allowed to interact for more time (Simkovic et al., 2018). Manipulations that explicitly test the effects of experimental design on nestmate discrimination would help to validate current knowledge, and could point toward the development of standard conditions, facilitating better comparison of results across studies.

One unanticipated finding was that introduced individuals that were smaller were antennated toward and possibly allogroomed at a higher rate than relatively larger introduced individuals. The smaller workers in the study are likely to have been younger termites and it has been shown that younger termites are at increased risk of disease compared to older termites (Rosengaus & Traniello, 2001). However, younger termites are also shown to be at lower risk when in the presence of older individuals (Rosengaus et al., 1998; Rosengaus & Traniello, 2001). The evidence that we find for this was not strong and the effect size was small, but we speculate that targeted allogrooming might be a potential link between these observations. Observations under more natural conditions would help to determine whether allogrooming of smaller individuals is a general phenomenon in termites and whether there is a potential role for this caste‐specific behavior in models of insect social immunity.

## 5 CONCLUSIONS

The results presented here demonstrate that nestmate discrimination is not constrained by shared broad‐scale environmental conditions—feeding on the same wood type—in single‐piece nesting termites. Accurate recognition of out‐group members is particularly important for single‐piece nesting termites and other organisms that experience frequent intergroup encounters, in which the outcome (and its fitness consequences) can vary. Our results support the idea that when groups frequently interact in a common environment, high‐resolution recognition cues, such as genetic cues, highly heterogeneous environmental factors, or their interaction, might be selected as more reliable indicators of group membership. Genetic or high‐resolution cues could be particularly important in species for which appropriate behavior toward out‐group members is context‐dependent and can change over time, for example, because of group fusion, as in single‐piece nesting termites, or because of fissioning events such as eviction or dispersal in some other social animals. Further experimental work will be important for understanding the potential mechanisms that organisms use to classify conspecifics in different contexts, particularly when there are important fitness consequences to incorrect decisions.

## AUTHOR CONTRIBUTIONS

**Rebecca F. B. Padget:** Conceptualization (equal); data curation (lead); formal analysis (lead); investigation (lead); methodology (equal); writing – original draft (lead); writing – review and editing (equal). **Michael A. Cant:** Conceptualization (equal); funding acquisition (equal); methodology (equal); project administration (supporting); supervision (equal); writing – review and editing (equal). **Faye J. Thompson:** Conceptualization (equal); funding acquisition (equal); methodology (equal); project administration (lead); supervision (equal); writing – original draft (supporting); writing – review and editing (equal).

## 7 FUNDING INFORMATION

This work was supported by a Natural Environment Research Council grant number: NE/S000046/1, awarded to F.J.T. and M.A.C.

## Supporting information


Appendix S1

## Data Availability

Data used in this paper will be available on FigShare on acceptance of the manuscript.
