# Supplementary material for: Us, them, and the others: Testing for discrimination amongst outgroups in a single‐piece nesting termite, Zootermopsis angusticollis
Source: Ecol Evol. 2023 Mar 21;13(3):e9901. doi: 10.1002/ece3.9901 (PMC10030232; doi:10.1002/ece3.9901)
Supplement: Supplementary file 1 — Appendix S1 [file ECE3-13-e9901-s001.docx]

**Supplementary Information**

**Inbreeding analysis**

These analyses were conducted to test the robustness of our results given the unknown factors about the potential genetic components of nestmate recognition cues in our experiment. Because only 4 (known) replicates used individuals that shared a grandparent, we could not establish whether there was an effect of relatedness between the newcomer and the group into which it was introduced. We did however conduct an analysis to determine whether there was an effect of inbreeding status, a proxy for within-group relatedness. We were able to include inbreeding status (whether the alates that founded the incipient colony used were from the same stock colony – i.e. were inbred – or not – i.e. were outbred) for 32 incipient colonies involved in a total of 43 replicates. This reduction in sample size reduces the statistical power of the models, particularly because the sample size was already relatively small. We must therefore be cautious in interpreting these results, which have wide uncertainty.

For antennation and recoiling, we find evidence against an effect of inbreeding status on behaviour. For allogrooming, there is weak evidence for an effect of inbreeding status. We report these results in the Supplementary Information because: power is reduced in these models due to lack of data; there is no evidence of an effect; and because we feel it is important to distinguish these exploratory analyses from the analyses for which we had a priori predictions, given in the main text.

**Statistical methods**

For the three behaviours that we analysed (allogrooming, antennation and recoiling), we added an additional fixed effect term for inbreeding status to the models described in the main text. Inbreeding status was a two-level factor indicating whether the alates had come from the same stock colony (i.e. were inbred), or a different stock colony (i.e. were outbred). 24 of the 39 incipient colonies used were inbred, 8 were outbred and 7 had unknown inbreeding status (one or both alates were from unknown stock colonies). Of the 54 pairings. 33 involved a newcomer being introduced to members of an inbred colony; 10 an outbred colony; and 11 were unknown. We removed the unknowns from the additional inbreeding analysis, reducing the sample size to 43. To determine whether inbreeding had an effect on the behaviour, we calculated the Bayes factor of the model including inbreeding status over the model without inbreeding status after re-running the model without inbreeding status so that both models were run on the same dataset to ensure that marginal likelihoods were comparable for Bayes factor calculation. We report the Bayes factor of the model including inbreeding status over the model without inbreeding status and where there was evidence supporting an effect of inbreeding, we report the inclusion Bayes factors and effect sizes from this model.

**Results from models including inbreeding as a predictor**

***Allogrooming***

We found weak Bayes factor evidence that the model including inbreeding status is a better descriptor of the allogrooming data than the model without inbreeding status (Bayes factor = 1.5). We therefore used models containing inbreeding status to calculate new inclusion Bayes factors for the other parameters in the model to determine whether the inclusion of inbreeding status affects our conclusions about the other predictors in the model. Inclusion Bayes factors from the new models are given in Table 1.

Table 1: Original inclusion Bayes factors and effect sizes from the model without inbreeding status compared to inclusion Bayes factors and effect sizes from model including inbreeding status for allogrooming. Bold results indicate a potential qualitative change in result.

| **Predictor** | **BF_inclusion_, with inbreeding status** | **BF_inclusion_, without inbreeding status** | **Effect size, with inbreeding status** | **Effect size, without inbreeding status** | **Qualitative interpretation** |
| --- | --- | --- | --- | --- | --- |
| Newcomer identity:wood type interaction | **0.44** | **0.26** | **0.016** | **0.25** | There was **moderate** evidence against an effect; there is now **weak** evidence against an effect. |
| Newcomer identity | **1.7** | **0.36** | **0.12** | **0.69** | There was weak evidence **against** an effect; there is now weak evidence **for** an effect (but the effect size is smaller). |
| Relative size | **0.84** | **3.1** | **-1.12** | **-1.9** | There was moderate evidence **for** an effect; there is now weak evidence **against** an effect. |
| Inbreeding status | 1.5 | NA |  | NA | Weak evidence for an effect. |

***Antennation***

There is “extreme” evidence that the model not including inbreeding status was the better fitting model (Bayes factor = 212321 in favour of model without inbreeding status). We therefore did not continue with further analysis.

***Recoiling***

There is weak evidence that the model not including inbreeding status was the better fitting model (Bayes factor = 2.1 in favour of model without inbreeding status). We therefore did not continue with further analysis.
